# Ube2s-stabilized β-catenin protects against myocardial ischemia/reperfusion injury by activating HIF-1α signaling

**DOI:** 10.18632/aging.102960

**Published:** 2020-04-06

**Authors:** Xi Chen, Chiyao Wang, Pei Yang, Lei Shi, Haiyan Wang

**Affiliations:** 1 Department of Pharmacy, The Second Affiliated Hospital of Air Force Medical University, Xi’an, China; 2 Department of Cardiology, The Second Affiliated Hospital of Air Force Medical University, Xi’an, China

**Keywords:** Ube2s, β-catenin, myocardial ischemia/reperfusion injury, HIF-1α, ubiquitination

## Abstract

The activation of hypoxia-inducible factor (HIF) is an important event for mediating the adaptive response to myocardial ischemia/reperfusion (MI/R) injury. The ubiquitin-conjugating enzyme E2S (Ube2s) catalyzes ubiquitin conjugation to target proteins. Here, we report the positive regulation of HIF-1α signaling by Ube2s via stabilizing β-catenin, by which Ube2s acts to protect against MI/R injury. We show that Ube2s expression is upregulated in the hearts of mice subjected to MI/R injury. Functionally, Ube2s depletion exacerbates and its overexpression ameliorates MI/R injury. In addition, Ube2s augments the activation of HIF-1α and reduces myocardial apoptosis. Moreover, Ube2s induces the accumulation of β-Catenin through increasing its stabilization. Importantly, β-Catenin knockdown abrogates Ube2s-augmented HIF-1α activation, and meanwhile, diminishes the protective effect of Ube2s on MI/R injury, thus establishing a causal link between Ube2s-stabilized β-catenin and HIF-1α-mediated myocardial protection. Altogether, this study identifies the Ube2s/β-catenin/HIF-1α axis as a novel protective regulator involved in MI/R injury, and also implies that it might represent a potential therapeutic target for ameliorating MI/R injury.

## INTRODUCTION

Myocardial infarction (MI) is a common cause of death and disability across the world, for which the most effective therapeutic intervention is timely myocardial reperfusion [1]. However, reperfusion itself causes further death of vulnerable cardiomyocytes, known as myocardial ischemia/reperfusion (MI/R) injury [2]. Currently, there is still no effective treatment for reducing MI/R injury, which compromises the therapeutic effectiveness in patients with MI [1, 3]. Although it has long been recognized that the pathogenesis of MI/R injury is multifactorial and the myocardial apoptosis is a major pathogenic factor [4–6], the regulatory mechanisms leading to myocardial apoptosis are still not fully understood. Further studies are needed to pursue novel therapeutic targets and strategies for reducing myocardial apoptosis, whereby preventing or minimizing MI/R injury.

On the other hand, accumulating evidence has suggested that myocardial cell survival during MI/R injury could be improved by modulating the expression of genes participating in promoting glycolysis, reducing ROS production and proapoptotic protein expression and limiting mitochondrial metabolism [7]. The hypoxia-inducible factor (HIF) belongs to a class of transcription factors that function to regulate the expression of nearly 200 downstream target genes, which coordinates a cellular adaptive response to hypoxia and/or ischemia [7, 8], and therefore the activation of HIF plays a protective role against the consequences of oxygen deprivation [9]. Moreover, several studies have reported that HIF-1α is activated in ischemic myocardium, and that the HIF-dependent program of gene expression enhances cell survival during MI/R injury, indicating that HIF activation confers protection against MI/R injury [10–12]. For instance, HIF-1α protects the heart against MI/R injury by promoting aerobic glycolysis and decreasing oxidative stress via upregulating its target genes, including HO-1, PDK-1, VEGF and HK-2 [12].

The ubiquitin-conjugating enzyme E2S (Ube2s) is a K11 linkage-specific E2, which catalyzes the elongation of K11-linked polyubiquitin chain on substrates to promote proteasome-mediated degradation [13]. However, recently, Ube2s has been associated with the stabilization of β-Catenin via ubiquitination modification [14]. Additionally, β-Catenin was found to interact with HIF-1 to promote cellular adaptation to hypoxia [15]. Furthermore, β-catenin protects against hepatic I/R injury in mice through augmenting HIF-1 signaling [16]. Given these clues, we hypothesized that Ube2s may play a role in MI/R injury. In this study, we report the protection of Ube2s against MI/R injury in a mouse model, during which the excessive activation of HIF-1α signaling induced by Ube2s-stabilized β-catenin constitutes a critical mechanism.

## RESULTS

### Ube2s expression is upregulated in the heart after MI/R injury

**Figure 1:**
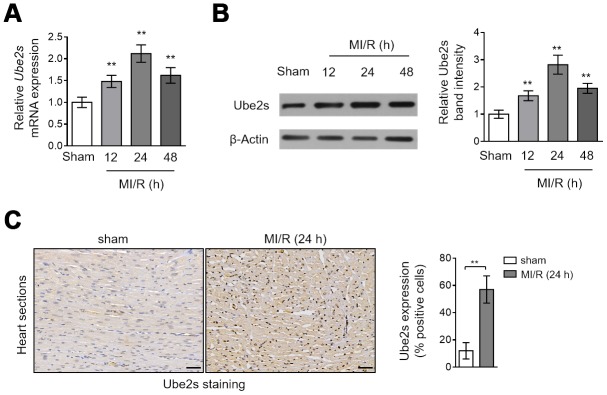
**Ube2s expression is upregulated after MI/R injury.** (**A**) qRT-PCR analysis of the mRNA level of Ube2s in the heart from C57BL/6 mice following 12 h, 24 h and 48 h of MI/R injury. Samples from mice receiving sham surgery were used as controls. Each group includes 8 mice. The results were normalized to β-Actin and expressed as relative to sham group. Data are mean ± SD. Data were compared with sham group using one-way ANOVA analysis. **, P < 0.01. (**B**) Western blotting analysis of the protein level of Ube2s in the heart as described in (**A**). β-Actin was used as a loading control. The representative band images are presented (left). The analysis of the relative band intensity is also presented (right). Data are mean ± SD. Data were compared with sham group using one-way ANOVA analysis. **, P < 0.01. (**C**) Immunohistochemistry analysis of Ube2s expression in the heart from mice subjected to sham or MI/R injury for 24 h as descried in (**A**). The representative images are shown (left). The analysis of percentage of positive stained cells is also depicted (right). Scale bar, 50 μm. Data are mean ± SD. Data were compared with sham group using Student’s *t*-test. **, P < 0.01.

Although the well-established role of Ube2s is cell cycle regulation [13, 17, 18], it was recently reported that Ube2s contains hypoxia-response elements in promoter region and its expression can be induced under hypoxia conditions [19]. Yet, as far as we know, what change Ube2s expression would display after MI/R injury is not investigated. To address it, we compared its expression in the heart tissues collected from mice subjected to sham or MI/R injury. The results obtained from the quantitative reverse transcription PCR (qRT-PCR) analysis showed that compared with sham operation, the transcript level of Ube2s in the heart tissues was increased and peaked at 24 h after MI/R injury (Figure 1A, Supplementary Figure 1A). In addition, similar results could be obtained when comparing Ube2s protein level between sham and MI/R groups through Western blotting analysis (Figure 1B, Supplementary Figure 1B). Moreover, the immunohistochemical staining analysis of the heart sections showed that Ube2s expression was enhanced after MI/R injury (Figure 1C). Taken together, these results indicate that Ube2s expression is upregulated in the heart at both transcript and protein levels after MI/R injury in this mouse model.

### Ube2s protects against MI/R injury

**Figure 2:**
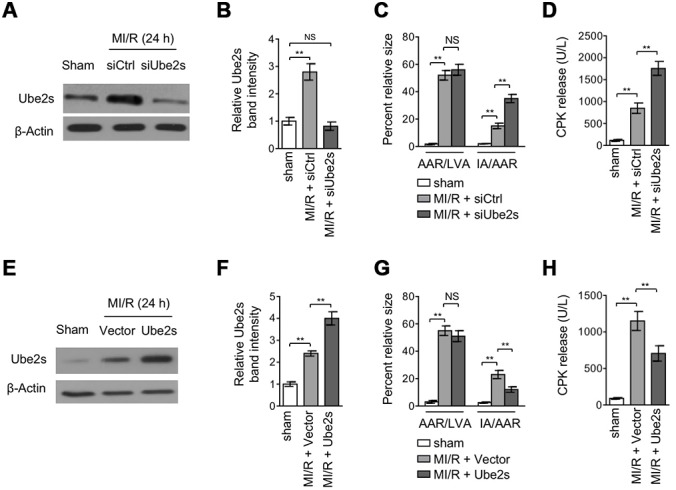
**Ube2s acts to protect against MI/R injury.** (**A**–**B**) C57BL/6 mice were intra-myocardially transfected with control siRNA (siCtrl) or Ube2s siRNA (siUbe2s) 48 h prior to MI/R surgery. Following 24 h of reperfusion, the protein level of Ube2s in the heart was analyzed by Western blotting. Samples from sham group were used as controls. Each group includes 8 mice. β-Actin was used as a loading control. The representative band images (**A**) and relative band intensity analysis (**B**) are presented. Data are mean ± SD. Data were compared with sham group using Student’s *t*-test. **, P < 0.01; NS, not significant. (**C**) Heart samples were harvested as described in (**A**), and the mid-myocardial cross sections were prepared. The infarct size in the heart sections was quantified, and the results of percentage of size are shown. AAR/LVA, ratio of area at risk (AAR) to left ventricular area (LVA); IA/AAR, ratio of infarct area (IA) to AAR. Data are mean ± SD. Data were compared using Student’s *t*-test. **, P < 0.01; NS, not significant. (**D**) C57BL/6 mice were treated as in (**A**). The samples of serum were collected and the level of creatine phosphokinase (CPK) was quantified. Data are mean ± SD. Data were compared using Student’s *t*-test. **, P < 0.01. (**E**, **F**) C57BL/6 mice were intra-myocardially infected with lentivirus expressing vector control or Ube2s 48 h prior to MI/R surgery. Following 24 h of reperfusion, the protein level of Ube2s in the heart was analyzed by Western blotting. Samples from sham group were used as controls. Each group includes 8 mice. β-Actin was used as a loading control. The representative band images (**E**) and relative band intensity analysis (**F**) are presented. Data are mean ± SD. Data were compared using Student’s t-test. **, P < 0.01. (**G**) Heart samples were harvested as described in (**E**), and the mid-myocardial cross sections were prepared. The infarct size in the heart sections was quantified, and the results of percentage of size are shown. AAR/LVA, ratio of area at risk (AAR) to left ventricular area (LVA); IA/AAR, ratio of infarct area (IA) to AAR. Data are mean ± SD. Data were compared using Student’s *t*-test. **, P < 0.01; NS, not significant. (**H**) C57BL/6 mice were treated as in (**E**). The samples of serum were collected and the level of creatine phosphokinase (CPK) was quantified. Data are mean ± SD. Data were compared using Student’s *t*-test. **, P < 0.01.

The upregulation of Ube2s expression after MI/R injury implies that it may play a functional role in this pathological condition. To test this possibility, we performed Ube2s expression knockdown in the mouse heart using the technique of siRNA transfection in vivo [20]. Western blotting analysis revealed that the upregulated Ube2s expression in the heart after MI/R injury was completely abrogated by the transfection of siRNA targeting the mouse Ube2s (Figure 2A–2B), indicating a high efficacy of siRNA-mediated knockdown in vivo. Next, to reflect the effect of Ube2s knockdown on MI/R injury, the mouse heart slices were stained with Evans blue and 2,3,5-triphenyltetrazolium chloride (TTC) to delineate the area at risk (AAR) and infarct area (IA), respectively [21]. The quantification analysis showed that although the ratio of AAR to left ventricular area (LVA) in siUbe2s group had no statistical significance when comparing with that of siCtrl, the ratio of IA to AAR was significantly higher in siUbe2s group (Figure 2C), suggesting that Ube2s expression knockdown increases myocardial infarct size after MI/R injury. Consistently, the detrimental effect of Ube2s knockdown after MI/R injury was reinforced by the observation that the released level of serum creatine phosphokinase (CPK) was prominently higher in mice transfected with siUbe2s (Figure 2D), which indicates an enlarged myocyte injury in the absence of Ube2s after MI/R injury. These data demonstrate that Ube2s depletion in the heart exacerbates MI/R injury.

The effects of Ube2s expression knockdown shown above conversely imply that Ube2s per se may play a protective role against MI/R injury. To verify this, we overexpressed Ube2s via lentivirus infection in the heart after MI/R injury [22]. The overexpression of Ube2s in the heart from mice with MI/R injury was confirmed by Western blotting analysis (Figure 2E–2F). Importantly, in agreement with the results from Ube2s knockdown (Figure 2C–2D), Ube2s overexpression instead resulted in a lower ratio of IA to AAR (Figure 2G) as well as a reduced level of released serum CPK (Figure 2H) after MI/R injury, as compared with empty vector control. Thus, these lines of evidence together show that Ube2s in the heart functions to protect against MI/R injury.

### Ube2s augments HIF-1α activation and decreases apoptosis after MI/R injury

**Figure 3:**
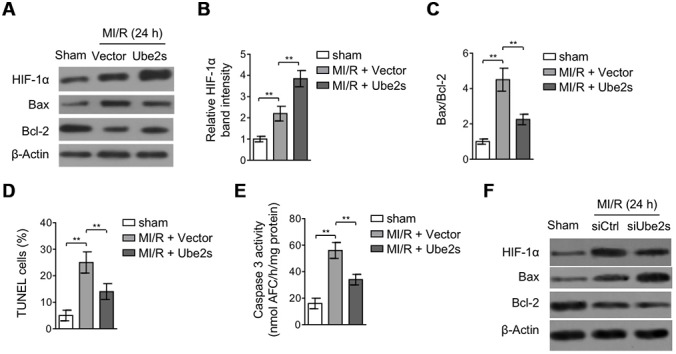
**Ube2s augments HIF-1α activation and decreases apoptosis after MI/R injury.** (**A**–**C**) C57BL/6 mice were intra-myocardially infected with lentivirus expressing vector control or Ube2s 48 h prior to MI/R surgery. Following 24 h of reperfusion, the protein level of HIF-1α, Bax and Bcl-2 in the heart was analyzed by Western blotting. Samples from sham group were used as controls. Each group includes 8 mice. β-Actin was used as a loading control. The representative band images (**A**) and relative band intensity analysis of HIF-1α (**B**) and ratio of Bax/Bcl-2 (**C**) are presented. Data are mean ± SD. Data were compared using Student’s *t*-test. **, P < 0.01. (**D**) Heart samples were harvested as described in (**A**), and heart sections were prepared. The apoptosis was detected using TUNEL staining. The statistical analysis of percentage of TUNEL positive cells is shown. Data are mean ± SD. Data were compared using Student’s *t*-test. **, P < 0.01. (**E**) Heart samples were harvested as described in (**A**). The supernatants of the homogenized heart samples were collected, and the caspase-3 activity was determined. The results are expressed as the nmol AFC/h/mg protein. Data are mean ± SD. Data were compared using Student’s *t*-test. **, P < 0.01. (**F**) C57BL/6 mice were intra-myocardially transfected with control siRNA (siCtrl) or Ube2s siRNA (siUbe2s) 48 h prior to MI/R surgery. Following 24 h of reperfusion, the protein level of HIF-1α, Bax and Bcl-2 in the heart was analyzed by Western blotting. Samples from sham group were used as controls. Each group includes 8 mice. β-Actin was used as a loading control. The representative band images are presented.

Among the protective mechanisms against MI/R injury, the adaptive response in the heart initiated by the activation of transcriptional complex hypoxia-inducible factor 1α (HIF-1α) has emerged as a key cardioprotective factor for mediating the survival of cardiomyocytes [10, 11, 23]. Lately, Ube2s was found to have a positive correlation with HIF-1α signaling in renal and cervical cancer cells [19, 24]. Therefore, to elucidate the mechanism by which Ube2s protects against MI/R injury, we tended to investigate whether HIF-1α is involved in Ube2s-mediated effects. To this end, we first compared the expression of HIF-1α in the heart when Ube2s was overexpressed or not. Expectedly, the results showed that HIF-1α expression was upregulated after MI/R injury compared with sham operation (Figure 3A–3B). Remarkably, the upregulation of HIF-1α expression was further enhanced when Ube2s was overexpressed in the heart (Figure 3A–3B), suggesting that Ube2s augments HIF-1α activation after MI/R injury. Functionally, coinciding with the cardioprotection exhibited by HIF-1α activation after MI/R injury [25, 26], Ube2s-augmented HIF-1α activation was accompanied by the reduced apoptosis of cardiomyocytes, as evidenced by reduced expression of Bax and increased expression of Bcl-2 (Figure 3A, Figure 3C). Furthermore, analyses of TUNEL staining of heart slices (Figure 3D) and caspase 3 activity measurement using the heart lysates (Figure 3E) showed that the apoptosis of cardiomyocytes was indeed suppressed by Ube2s overexpression. Conversely, Ube2s knockdown diminished the activation of HIF-1α, and meanwhile, promoted the apoptosis of cardiomyocytes (Figure 3F). Collectively, it could be concluded that reducing apoptosis of cardiomyocytes at least in part accounts for the protective role of Ube2s against MI/R injury, which may be associated with the augmented HIF-1α activation.

### HIF-1α activation contributes to Ube2s protective effect against cardiomyocyte apoptosis and MI/R injury

**Figure 4:**
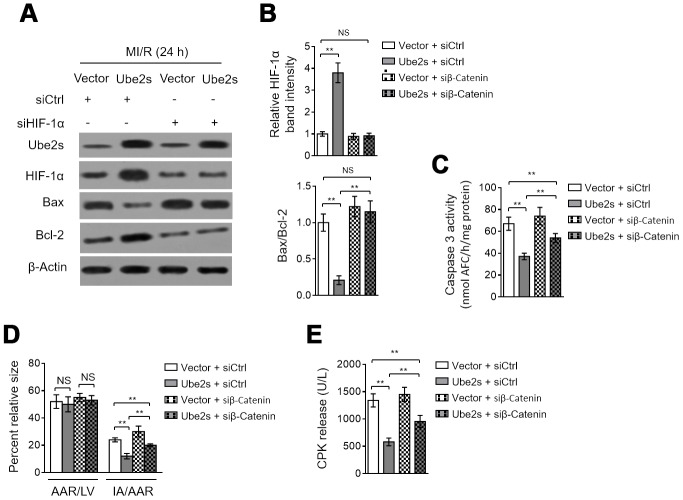
**HIF-1α activation mediates Ube2s role in cardiomyocyte apoptosis and MI/R injury.** (**A**–**C**) C57BL/6 mice were intra-myocardially infected with lentivirus expressing vector control or Ube2s in the presence or absence of transfection of control siRNA (siCtrl) or HIF-1α siRNA (siHIF-1α) 48 h prior to MI/R surgery. Following 24 h of reperfusion, the protein level of Ube2s, HIF-1α, Bax and Bcl-2 in the heart was analyzed by Western blotting. Samples from sham group were used as controls. Each group includes 8 mice. β-Actin was used as a loading control. The representative band images (**A**) and relative band intensity analysis of HIF-1α and ratio of Bax/Bcl-2 (**B**) are presented. (**C**) The supernatants of the homogenized heart samples were collected, and the caspase-3 activity was determined. The results are expressed as the nmol AFC/h/mg protein. Data are mean ± SD. Data were compared using Student’s *t*-test. **, P < 0.01; NS, not significant. (**D**) Heart samples were harvested as described in (**A**), and the mid-myocardial cross sections were prepared. The infarct size in the heart sections was quantified, and the results of percentage of size are shown. AAR/LVA, ratio of area at risk (AAR) to left ventricular area (LVA); IA/AAR, ratio of infarct area (IA) to AAR. Data are mean ± SD. Data were compared using Student’s *t*-test. **, P < 0.01; *, P < 0.05; NS, not significant. (**E**) C57BL/6 mice were treated as in (**A**). The samples of serum were collected and the level of creatine phosphokinase (CPK) was quantified. Data are mean ± SD. Data were compared using Student’s *t*-test. **, P < 0.01.

We asked whether HIF-1α activation mediates Ube2s function in MI/R injury. As shown, HIF-1α knockdown via siRNA transfection significantly reversed the Ube2s-induced reduction of both Bax/Bcl-2 (Figure 4A–4B) and caspase 3 activity (Figure 4C) in the heart after MI/R injury, although not totally recovered to that of vector control. These data describe that the anti-apoptotic effect of Ube2s on cardiomyocytes after MI/R injury at least partly relies on HIF-1α activation. To relate this finding to MI/R injury more closely, we checked the myocardial infarct size and serum CPK release. Indeed, in line with the results of cardiomyocyte apoptosis, HIF-1α knockdown markedly diminished the Ube2s-induced decline of IA/AAR (Figure 4D) and serum CPK release (Figure 4E). Hence, HIF-1α plays an important role in mediating Ube2s role for attenuating cardiomyocyte apoptosis and MI/R injury.

### Ube2s stabilizes β-Catenin after MI/R injury

**Figure 5:**
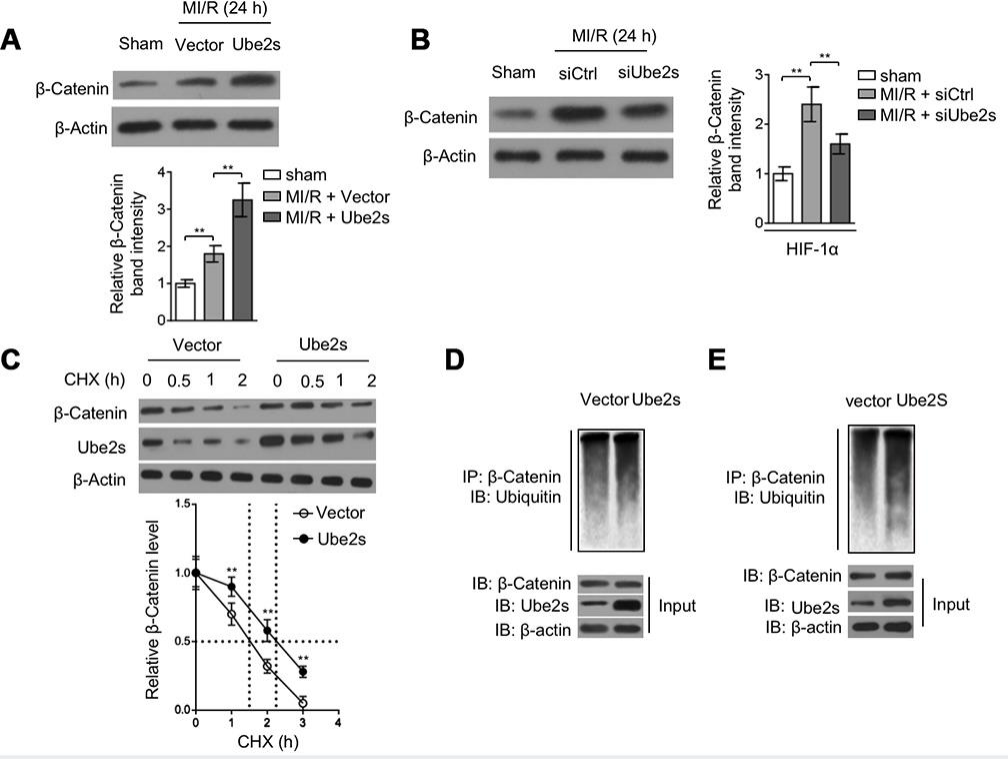
**Ube2s stabilizes β-Catenin after MI/R injury.** (**A**) C57BL/6 mice were intra-myocardially infected with lentivirus expressing vector control or Ube2s 48 h prior to MI/R surgery. Following 24 h of reperfusion, the protein level of β-Catenin in the heart was analyzed by Western blotting. Samples from sham group were used as controls. Each group includes 8 mice. β-Actin was used as a loading control. The representative band images (left) and relative band intensity analysis (right) are presented. Data are mean ± SD. Data were compared using Student’s *t*-test. **, P < 0.01. (**B**) C57BL/6 mice were intra-myocardially transfected with control siRNA (siCtrl) or Ube2s siRNA (siUbe2s) 48 h prior to MI/R surgery. Following 24 h of reperfusion, the protein level of β-Catenin in the heart was analyzed by Western blotting. Samples from sham group were used as controls. Each group includes 8 mice. β-Actin was used as a loading control. The representative band images (left) and relative band intensity analysis (right) are presented. Data are mean ± SD. Data were compared using Student’s *t*-test. **, P < 0.01. (**C**) The cardiomyocytes overexpressing empty vector or Ube2s were treated with cycloheximide (CHX) for increasing time periods as indicated. The protein expression of β-Catenin and Ube2s was determined by Western blotting analysis. β-Actin was used as a loading control. The representative band images (left) and relative band intensity analysis of β-Catenin (right) are presented. The half-life is depicted by dot line. Data are mean ± SD. Data were compared using Student’s *t*-test. **, P < 0.01. (**D**) The lysates of cardiomyocytes stably overexpressing empty vector or Ube2s were immunoprecipitated (IP) with β-Catenin antibody. The IP products were further analyzed by Western blotting to detect ubiquitin expression. The expression of β-Catenin and Ube2s in the input fraction is presented below. (**E**) The lysates of heart tissues from mice, intra-myocardially infected with lentivirus expressing vector control or Ube2s 48 h prior to MI/R surgery, were immunoprecipitated (IP) with β-Catenin antibody. The IP products were further analyzed by Western blotting to detect ubiquitin expression. The expression of β-Catenin and Ube2s in the input fraction is presented below.

Ube2s overexpression was found to increase HIF-1α stability [24, 27]. However, at present, the underlying mechanism is elusive. In a recent study, Ube2s has been demonstrated to induce the stabilization of β-Catenin via a mechanism of K11-linked polyubiquitination in mES cells [14]. Besides, previous studies have also shown that β-Catenin interacts with HIF-1α under hypoxic condition [15] and that β-catenin protects against hepatic I/R injury through augmented HIF-1α signaling [16]. These clues hint that β-Catenin may contribute to Ube2s-augmented HIF-1α. First, we tested whether Ube2s affects β-Catenin expression after MI/R injury. As shown in Figure 5A, compared with vector control, Ube2s overexpression in the heart after MI/R injury magnified the upregulation of β-Catenin. Conversely, Ube2s knockdown attenuated β-Catenin upregulation after MI/R injury (Figure 5B), suggesting that Ube2s expression has a positive correlation with β-Catenin level. Further, in cardiomyocyte cell line H9c2 cultured in vitro, cycloheximide (CHX) chasing experiment revealed that the half-life of β-Catenin was prolonged in cells overexpressed with Ube2s (Figure 5C). Moreover, similar results were obtained in primary cardiomyocytes (Supplementary Figure 2A). These observations suggest that Ube2s regulates β-Catenin level via promoting its stability. This notion is supported by the evidence that Ube2s overexpression increased the ubiquitination of β-Catenin in H9c2 cells (Figure 5D), which has been previously demonstrated that Ube2s-mediated ubiquitination of β-Catenin enhances its stability [14]. In concert, Ube2s-induced β-Catenin ubiquitination was also found when analyzing the heart tissues from mice (Figure 5E).

Of note, the stabilization of β-Catenin by Ube2s seems irrelevant to GSK-3β, an important upstream regulator [28], since we noticed that either Ube2s overexpression (Supplementary Figure 2B) or knockdown (Supplementary Figure 2C) did not obviously affect the phosphorylation level of GSK-3β. Furthermore, we found that in contrast to the wild-type Ube2s, the overexpression of C95S mutant of Ube2s, wherein the enzymatic activity is lost [13], could not promote the induced level of β-Catenin (Supplementary Figure 2D) or further increased the ubiquitination of β-Catenin (Supplementary Figure 2E) in the heart tissues after MI/R injury. Therefore, in addition to mES cells [14], Ube2s also serves to stabilize β-Catenin via ubiquitination modification in the heart after MI/R injury.

### β-Catenin knockdown abrogates Ube2s-augmented HIF-1α activation and diminishes protective effect on MI/R injury

**Figure 6:**
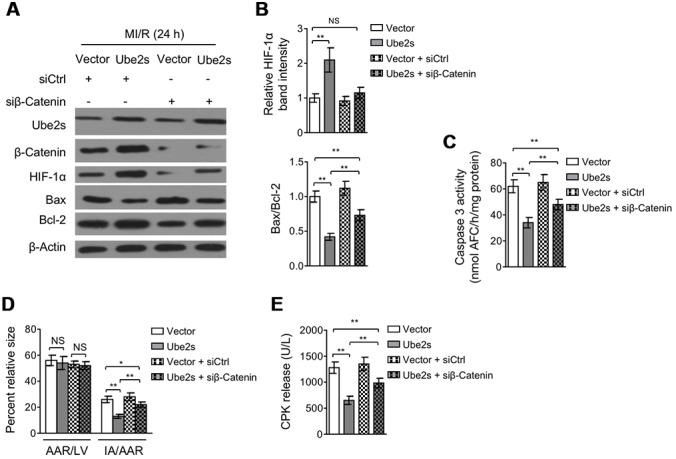
**β-Catenin knockdown abrogates Ube2s-augmented HIF-1α activation and diminishes Ube2s protective effect on MI/R injury.** (**A**–**B**) C57BL/6 mice were intra-myocardially infected with lentivirus expressing vector control or Ube2s in combination with the transfection with control siRNA (siCtrl) or β-Catenin siRNA (siβ-Catenin) 48 h prior to MI/R surgery. Following 24 h of reperfusion, the protein expression of targets as indicated in the heart was analyzed by Western blotting. Each group includes 8 mice. β-Actin was used as a loading control. The representative band images (**A**) and relative band intensity analysis (**B**) are presented. Data are mean ± SD. Data were compared using Student’s *t*-test. **, P < 0.01; NS, not significant. (**C**) Heart samples were harvested as described in (**A**). The supernatants of the homogenized heart samples were collected, and the caspase-3 activity was determined. The results are expressed as nmol AFC/h/mg protein. Data are mean ± SD. Data were compared using Student’s *t*-test. **, P < 0.01. (**D**) Heart samples were harvested as described in (**A**), and the mid-myocardial cross sections were prepared. The infarct size in the heart sections was quantified, and the results of percentage of size are shown. AAR/LVA, ratio of area at risk (AAR) to left ventricular area (LVA); IA/AAR, ratio of infarct area (IA) to AAR. Data are mean ± SD. Data were compared using Student’s *t*-test. **, P < 0.01; *, P < 0.01; NS, not significant. (**E**) C57BL/6 mice were treated as in (**A**). The samples of serum were collected and the level of creatine phosphokinase (CPK) was quantified. Data are mean ± SD. Data were compared using Student’s *t*-test. **, P < 0.01.

**Figure 7:**
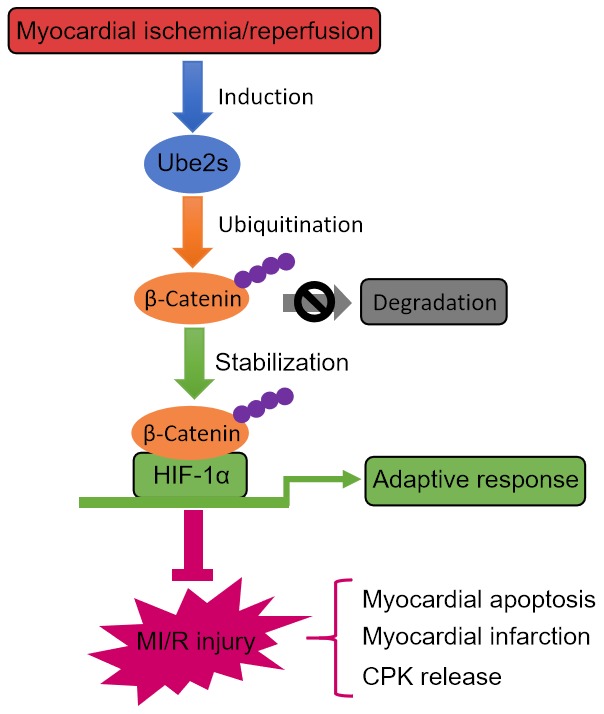
**Ube2s/β-catenin/HIF-1α axis protects against MI/R injury.** Graphic description of this study. Ube2s promotes HIF-1α activation through stabilizing β-catenin via a mechanism of ubiquitination modification, whereby acting to protect against MI/R injury, including reducing myocardial apoptosis, infarction and CPK release.

Lastly, we asked whether Ube2s-stabilized β-Catenin contributes to augmented HIF-1α activation and protective role of Ube2s against MI/R injury. For this purpose, we depleted β-Catenin in the heart using siRNA transfection. Western blotting analysis showed that Ube2s overexpression in the heart consistently resulted in enhanced expression of HIF-1α and β-Catenin after MI/R injury compared with vector control (Figure 6A–6B). However, when β-Catenin was depleted by siRNA transfection, the enhanced expression of HIF-1α by Ube2s overexpression was totally recovered to the level of vector control (Figure 6A–6B), illustrating that Ube2s-augmented HIF-1α activation strictly depends on the presence of β-Catenin. Functionally, along with the abrogated activation of HIF-1α, the decreased apoptosis of cardiomyocytes by Ube2s overexpression was substantially minimized, as shown by markedly recovered expression of Bax and Bcl-2 (Figure 6A–6B) and caspase 3 activity (Figure 6C). More importantly, consistent with the above attenuated cardioprotective effect, β-Catenin knockdown also diminished the alleviating effects of Ube2s overexpression on MI/R injury, as evidenced by the recovered ratio of IA to AAR (Figure 6D) as well as released level of serum CPK (Figure 6E). On the other hand, the C95S mutant of Ube2s, which is unable to promote β-Catenin stabilization via ubiquitination (Supplementary Figure 2C–2D), failed to upregulate β-Catenin level or induce the activation of HIF-1α (Supplementary Figure 3A–3B) after MI/R injury. Accordingly, the C95S mutant of Ube2s had no apparent effects on cardiomyocyte apoptosis (Supplementary Figure 3A–3C) or MI/R injury (Supplementary Figure 3D–3E), regardless of β-Catenin status. In conclusion, these data strongly indicate that Ube2s augments HIF-1α activation in the heart through stabilizing β-Catenin, which is a critical mechanism that underlies the protective role of Ube2s against MI/R injury (Figure 7).

## DISCUSSION

In addition to the upstream activated Wnt signaling that leads to the accumulation and nuclear translocation of β-Catenin, whereby it further activates the transcription complex T-cell factor/lymphoid enhancer factor (TCF/LEF) [29], several posttranslational modifications, such as phosphorylation, acetylation and ubiquitination of β-Catenin have also been described to profoundly influence its transcriptional activity [30]. However, different from its conventional role as a transcriptional activator for TCF/LEF, some studies have shown that β-Catenin can be diverted to adaptive signaling pathways including hypoxia inducible factor (HIF)-1 to corporately regulate the cellular response to hypoxia-induced oxidative stress [15, 31]. HIF-1 is composed of HIF-1α and HIF-1β. Under hypoxia, HIF-1α will be stabilized and then translocate to the nucleus and dimerize with HIF-1β, thus eventually binding to hypoxia response elements (HRE) for activating the transcription of target genes which are involved in modulating cellular metabolic changes [32]. Furthermore, it was recently reported that β-Catenin protects against hepatic I/R injury in mice through augmenting the HIF-1 signaling [16], offering an in vivo evidence demonstrating that β-Catenin could serve as a regulator involved in an effective tissue-specific response to I/R.

Given the above clues, our study was designed to investigate whether Ube2s, a K11 linkage-specific Ub-conjugating enzyme previously shown to regulate β-Catenin stabilization in embryonic stem (ES) cells [14], has a functional role in MI/R injury and further explore the underlying mechanism. We provide evidence showing that Ube2s is upregulated after MI/R injury, therefore firstly associating Ube2s with MI/R injury in a mouse model. Following functional studies show that Ube2s depletion exacerbates, and conversely, its overexpression ameliorates MI/R injury, as evidenced by infarct size and released level of serum CPK. In searching the mechanism that underlies Ube2s function in MI/R injury, we reveal that Ube2s augments the activation of HIF-1α and decreases myocardial apoptosis after MI/R injury, furthermore, Ube2s also functions to stabilize β-Catenin after MI/R injury, as it does in ES cells [14]. Lastly, the stabilized β-Catenin was demonstrated to be an important regulator that mediates the functional role of Ube2s after MI/R injury, as shown by the reverse proof that β-Catenin knockdown abrogates Ube2s-augmented HIF-1α activation and diminishes the protective effect on MI/R injury, and also evidenced by the failure of C95S mutant Ube2s in protecting against MI/R injury when the activation of β-Catenin/HIF-1α axis is not achieved. Thus, these results establish a causal link between Ube2s-stabilized β-Catenin and Ube2s-augmented HIF-1α activation as well as its protective effect on MI/R injury (Figure 7). In sum, our study identifies Ube2s as a novel regulator in the pathogenesis of MI/R injury, in which the cardioprotective function of the Ube2s/β-Catenin/HIF-1α axis may be related to the reduced cardiomyocyte apoptosis.

Ube2s expression is induced following MI/R injury at both transcript level and protein level, suggesting that the condition of MI/R injury may activate Ube2s transcription. Of note, the upregulated expression of Ube2s peaks at 24 h and then declines thereafter following MI/R injury, implying that there may exist a feed-back mechanism that controls Ube2s induction under this condition. Further studies are needed to discover how Ube2s expression is regulated at an extended period of time following MI/R injury. On the other hand, we speculate that the induced expression of Ube2s may be derived from the stimulation of hypoxia during the procedure of ischemia/reperfusion. This is very possible, because Ube2s gene promoter includes hypoxia-response elements, which have been demonstrated to be induced by hypoxia through HIF1α [19]. If this is the case, based on the regulation of HIF1α by Ube2s-stabilized β-Catenin, it could probably be further deduced that the crosstalk between Ube2s and HIF1α may engender a positive feedback loop, in which the level of HIF1α following MI/R injury is further increased (Figure 7). Given the protective role of HIF1α as revealed by our present study and others [33, 34], maintaining or even augmenting the Ube2s/HIF1α pathway may reduce the apoptosis of myocardiocytes through increasing their adaptive response to hypoxic pathogenic condition following MI/R injury, therefore holding potential therapeutic benefit in reducing infarct size and improving clinical outcome of patients with acute MI.

It’s well-established that the stabilization of β-Catenin by Ube2s is through modification of K11-linked polyubiquitination [14]. Consistently, we found that Ube2s overexpression increased the ubiquitination level of β-Catenin and elongated its half-life in cultured cardiomyocyte cell line H9c2. In addition, we also noticed that Ube2s overexpression increased and its depletion reduced β-Catenin expression in the heart tissue following MI/R injury, suggesting a positive correlation between Ube2s and β-Catenin, which we suppose is connected to the modification of polyubiquitination of β-Catenin, as we have proven that in the heart tissues following MI/R injury, Ube2s also promotes β-Catenin polyubiquitination. More importantly, we found that β-Catenin depletion abolished the enhanced expression of HIF-1α by Ube2s overexpression, however only partially recovered the protective effect of Ube2s following MI/R injury. These results clearly indicate that except for HIF-1α, other effectors downstream of Ube2s are responsible for mediating its function following MI/R injury. Elucidating this issue in the future may shed new light on how Ube2s participates in the pathogenesis of MI/R injury.

## MATERIALS AND METHODS

### Animals and MI/R model

All animal experiments were performed according to the protocols approved by The Second Affiliated Hospital of Air Force Medical University. Eight- to 10-week-old wild-type C57BL/6J male mice were purchased from the Jackson Laboratory and maintained in pathogen-free facilities and utilized for developing the MI/R model as reported in previous studies [21, 35]. Eight mice were allocated into each group prior to experiments based on body weight. Briefly, mice were initially anesthetized with sodium pentobarbital, and then the heart was exposed via an incision at the left chest. The myocardial infarction was developed through using a 6.0 silk suture slipknot ligated around the left anterior descending (LAD) of the coronary artery. The ischemic status was maintained for 30 min, followed by the reperfusion for 12 to 96 h according to experimental purposes. Meanwhile, the sham-operated mice underwent only the left thoracotomy were used as negative controls.

### Western blotting analysis

For extracting the total protein, the whole mouse heart tissues were minced and then lysed completely using the RIPA Lysis and Extraction Buffer (ThermoFisher Scientific, 89900) supplemented with the protease inhibitor cocktail (Roche, 04693132001). The supernatants of whole lysates were collected, and the protein concentration was quantified via the BCA Protein Assay Kit (Thermo Fisher Scientific, Inc., 23227). Protein samples were denatured for 5 min in SDS loading buffer at 100 °C. The Western blotting was conducted as described previously [36]. Briefly, protein samples were loaded and separated by SDS-PAGE and transferred to nitrocellulose filter (NC) membranes. After the block with 5% BSA, membranes were incubated overnight at 4 °C with primary antibodies against the following targets: Ube2s (Novus Biologicals, 1:500), HIF-1α (Invitrogen, 1:2000), Bax (Abcam, 1:1000), Bcl-2 (Abcam, 1:1000), β-Catenin (Cell Signaling, 1:500) and β-Actin (Santa Cruz, 1:5000). After the wash with TBST, membranes were incubated for 1 h at room temperature with corresponding secondary antibodies conjugated with HRP (Santa Cruz, 1:5000). The wash step was repeated, the protein bands were developed using the ECL Western Blotting Substrate (Pierce, 32106). The band intensity of target proteins was analyzed by ImageJ software.

### Quantitative reverse transcription PCR analysis

The total mRNA in the whole mouse heart tissues were isolated using the TRIzol reagent (ThermoFisher Scientific, 15596026), and the synthesis of cDNA was performed using the First Strand cDNA Synthesis Kit (OriGene, 11801-025) according to the manufacturer's instructions. The quantitative reverse transcription PCR (qRT-PCR) was conducted with cDNA template, QuantiTect SYBR Green PCR Kit (QIAGEN, 204141) and Real-Time PCR Detection System (Bio-Rad, CFX96 Touch). β-Actin was used as a reference control. The sequence of primers is accessible upon request.

### siRNA transfection and lentivirus infection in vivo

The knockdown of Ube2s and β-Catenin in mouse heart was performed using siRNA transfection in vivo. The specific siRNA targeting mouse Ube2s (siUbe2s) or β-Catenin (siβ-Catenin) (Invitrogen) or non-specific scrambled siRNA (siCtrl) (Invitrogen) were mixed with the in vivo-jetPEI reagent (Polyplus Transfection) and then delivered through intra-myocardial injections using the 32.5-gauge needle 48 h prior to MI/R surgery as performed previously [37]. The sequences of siRNAs targeting mouse genes are listed as follows: siCtrl GAACUGAUGACAGGGAGGCTT; siUbe2s CUGUCUCUAAGUUAUUUAAAU; siβ-Catenin UAGUCGUGGAAUAGCACCCUG. For Ube2s overexpression in the heart, a volume of 30 μl green fluorescent protein (GFP)-conjugated Ube2s lentivirus (wild-type or C95S mutant) or empty vector control lentivirus were injected into the left ventricle of mice 48 h prior to MI/R surgery as adapted to a previous study [38]. The knockdown and overexpression efficiency was confirmed by Western blot.

### Infarct size measurement

Following 24 h of reperfusion, 1% Evans blue (Sigma-Aldrich) was injected through the jugular vein into the aorta. The heart was then removed, washed with PBS and horizontally sectioned into 5-6 slices, which were stained for 15 min with 1 ml 1% 2, 3, 5-triphenyl tetrazolium chloride (TTC) (Sigma-Aldrich) at 37 °C. The slices were photographed with a photomicroscope. The size of left ventricular (LV), area at risk (AAR), and infarct area (IA) was quantified using ImageJ software.

### Myocyte injury evaluation

The myocyte injury was evaluated by the release level of serum creatine phosphokinase (CPK) as described before [21]. The blood of mice was obtained from the tail veins following 6 h of sham or MI/R surgery. After centrifugation, the serum was collected and the CPK activity was assessed using the Creatine Kinase (CK) Activity Colorimetric Assay Kit (BioVision, K777) at 450 nm absorbance monitored by the Spectra Max-Plus microplate spectrophotometer (Molecular Devices) according to the manufacturer’s instructions.

### TUNEL staining

The apoptosis was measured by TUNEL positive cells stained using the In Situ Cell Death Detection Kit (Roche, 11684817910) following the manufacturer’s instructions. In brief, the hearts of sham or MI/R mice were perfused with ice-cold PBS and fixed with 4% paraformaldehyde (PFA). The hearts were embedded in paraffin and cut into 5 μm thick sections. Each section was stained with TUNEL reaction reagents in the dark and finally counterstained with DAPI. The images were captured under the A1 laser scanning confocal microscopy (Nikon). TUNEL positive cells were counted at 5 random fields of 3 different sections. The results are expressed as the percentage of TUNEL positive cells among total cells in the field, which were stained blue with DAPI staining.

### Caspase-3 activity measurement

The apoptosis was reflected by the caspase-3 activity measured using the Ac-DEVD-AFC Caspase-3 Fluorogenic Substrate (BD Pharminge, 556574) according to the manufacturer’s instructions. Briefly, heart tissues from sham or MI/R mice were lysed on ice. After centrifugation, the supernatants were collected and the protein concentration was quantified using BCA method. A total of 50 μg proteins were pipetted and mixed with assay buffer supplemented with 10 mM dithiothreitol (DTT). The fluorescence emission of the AFC (400 nm) was measured via the Spectra Max-Plus Microplate Spectrophotometer (Molecular Devices). The caspase-3 activity is expressed as nmol AFC/h/mg protein.

### Culture and treatment of cardiomyocytes

The cardiomyocyte cell line H9c2 was purchased from the American Type Culture Collection (Manassas, VA). H9c2 cells were cultured in complete Dulbecco’s modified Eagle’s medium (Invitrogen) supplemented with 10% fetal bovine serum (Invitrogen) and 50 U/mL penicillin/streptomycin (Invitrogen) at 37 °C in a humidified atmosphere with 5% CO_2_. For Ube2s overexpression, H9c2 cells were transfected with plasmids using the Lipofectamine 2000 (Invitrogen) based on the manufacturer's protocol. After 2 days, cycloheximide (CHX) chasing assay was then performed with H9c2 cells treated with or without 50 μM CHX for increasing time periods.

### Statistics

Data are expressed as the mean ± SD. The data between two groups were analyzed by unpaired Student’s *t*-test. The data among more than two groups were compared by one-way analysis of variance (ANOVA). The values with *P* < 0.05 were defined as statistically significant.

## Supplementary Material

Supplementary Figures
