# Supplementary material for: Ube2s-stabilized β-catenin protects against myocardial ischemia/reperfusion injury by activating HIF-1α signaling
Source: Aging (Albany NY). 2020 Apr 6;12(7):5716–32. doi: 10.18632/aging.102960 (PMC7185123; doi:10.18632/aging.102960)
Supplement: Supplementary Figures [file aging-12-102960-s001..pdf]

SUPPLEMENTARY FIGURES

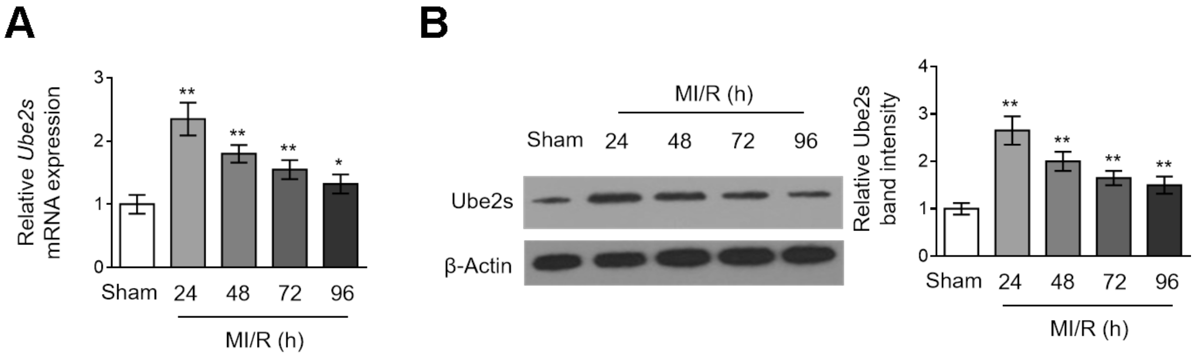

Supplementary Figure 1. Ube2s expression is upregulated after MI/R injury. (**A**) qRT-PCR analysis of the mRNA level of Ube2s in the heart from C57BL/6 mice following 24 h, 48 h, 72 h and 96 h of MI/R injury. Samples from mice receiving sham surgery were used as controls. Each group includes 8 mice. The results were normalized to β-Actin and expressed as relative to sham group. Data are mean ± SD. Data were compared with sham group using one-way ANOVA analysis. **, P < 0.01; *, P < 0.05. (**B**) Western blotting analysis of the protein level of Ube2s in the heart as described in (**A**). β-Actin was used as a loading control. The representative band images are presented (left). The analysis of the relative band intensity is also presented (right). Data are mean ± SD. Data were compared with sham group using one-way ANOVA analysis. **, P < 0.01.

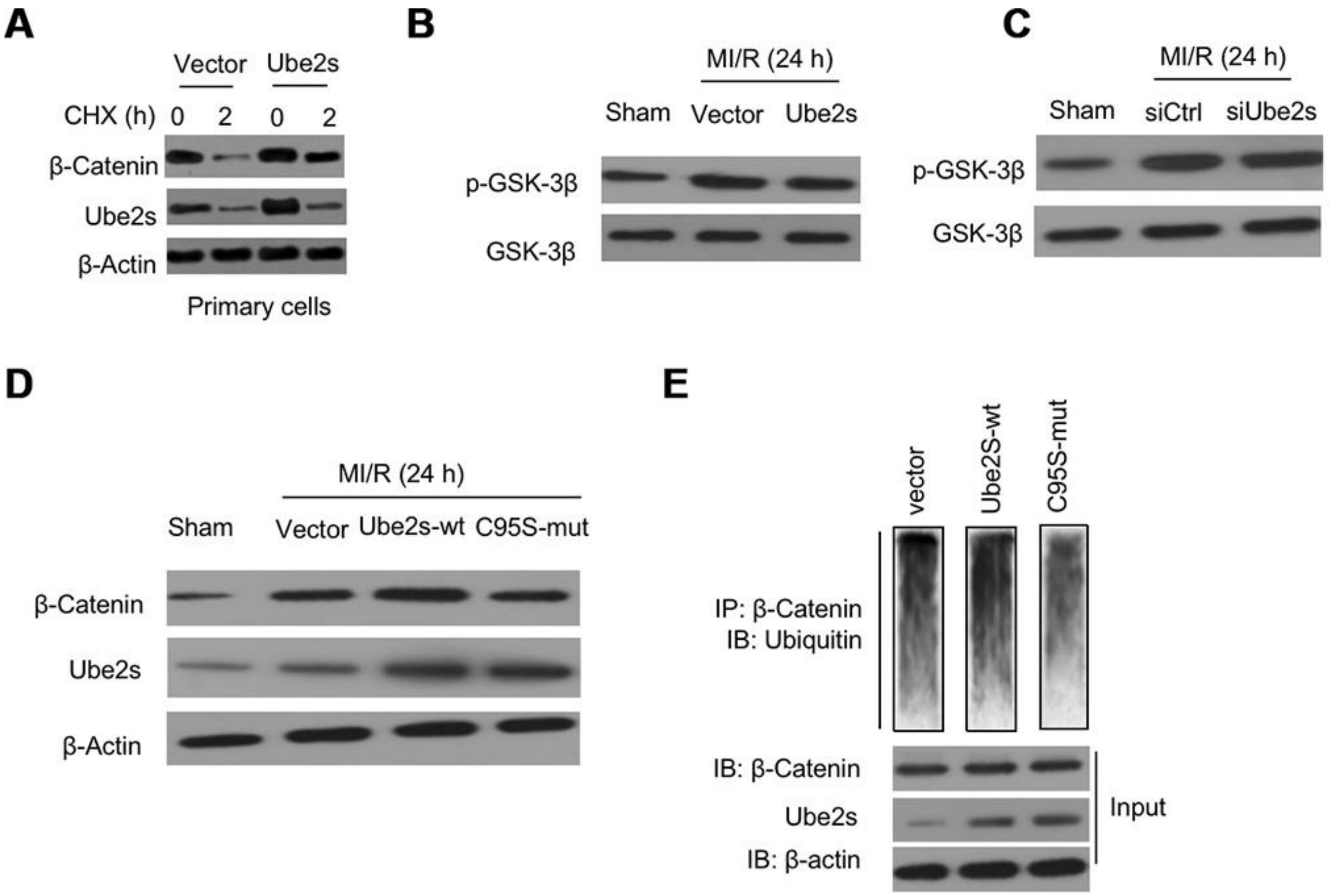

Supplementary Figure 2. Ube2s stabilizes β-Catenin. (**A**) The primary cardiomyocytes overexpressing empty vector or Ube2s were treated with CHX for 2 h. The protein expression of β-Catenin and Ube2s was determined by Western blotting analysis. β-Actin was used as a loading control. The representative band images are presented. (**B**) C57BL/6 mice were intra-myocardially infected with lentivirus expressing vector control or Ube2s 48 h prior to MI/R surgery. Following 24 h of reperfusion, the protein level of GSK-3β and p-GSK-3β in the heart was analyzed by Western blotting. Samples from sham group were used as controls. Each group includes 8 mice. The representative band images are presented. (**C**) C57BL/6 mice were intra-myocardially transfected with control siRNA (siCtrl) or Ube2s siRNA (siUbe2s) 48 h prior to MI/R surgery. Following 24 h of reperfusion, the protein level of GSK-3β and p-GSK-3β in the heart was analyzed by Western blotting. Samples from sham group were used as controls. Each group includes 8 mice. The representative band images are presented. (**D**) C57BL/6 mice were intra-myocardially infected with lentivirus expressing vector control or wild-type Ube2s (Ube2s-wt) or C95S mutant form of Ube2s (C95S-mut) 48 h prior to MI/R surgery. Following 24 h of reperfusion, the protein level of β-Catenin and Ube2s in the heart was analyzed by Western blotting. Samples from sham group were used as controls. Each group includes 8 mice. The representative band images are presented. (**E**) The lysates of heart tissues from mice as described in (**D**) were immunoprecipitated (IP) with β-Catenin antibody. The IP products were further analyzed by Western blotting to detect ubiquitin expression. The expression of β-Catenin and Ube2s in the input fraction is presented below.

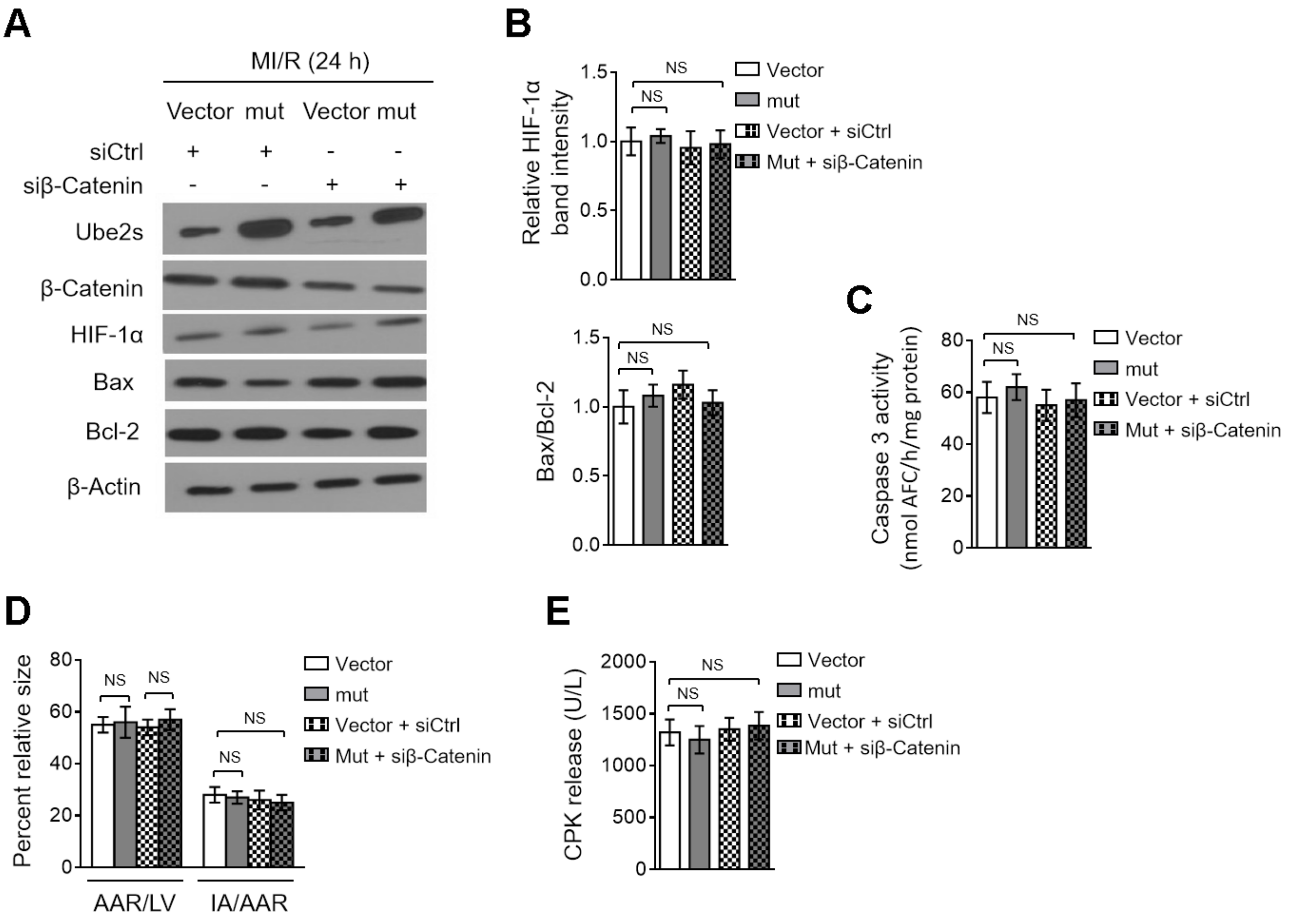

Supplementary Figure 3. C95S mutant Ube2s fails to activate β-Catenin/HIF-1α axis and influence MI/R injury. (**A**–**B**) C57BL/6 mice were intra-myocardially infected with lentivirus expressing vector control or C95S mutant Ube2s (mut) in combination with the transfection with control siRNA (siCtrl) or β-Catenin siRNA (siβ-Catenin) 48 h prior to MI/R surgery. Following 24 h of reperfusion, the protein expression of targets as indicated in the heart was analyzed by Western blotting. Each group includes 8 mice. β-Actin was used as a loading control. The representative band images (**A**) and relative band intensity analysis (**B**) are presented. Data are mean ± SD. Data were compared using Student's t-test. NS, not significant. (**C**) Heart samples were harvested as described in (**A**). The supernatants of the homogenized heart samples were collected, and the caspase-3 activity was determined. The results are expressed as nmol AFC/h/mg protein. Data are mean ± SD. Data were compared using Student's t-test. NS, not significant. (**D**) Heart samples were harvested as described in (**A**), and the mid-myocardial cross sections were prepared. The infarct size in the heart sections was quantified, and the results of percentage of size are shown. AAR/LVA, ratio of area at risk (AAR) to left ventricular area (LVA); IA/AAR, ratio of infarct area (IA) to AAR. Data are mean ± SD. Data were compared using Student's t-test. NS, not significant. (**E**) C57BL/6 mice were treated as in (**A**). The samples of serum were collected and the level of creatine phosphokinase (CPK) was quantified. Data are mean ± SD. Data were compared using Student's t-test. NS, not significant.
